# Design and management of humanitarian supply chains: challenges, solutions, and frameworks

**DOI:** 10.1007/s10479-022-05021-7

**Published:** 2022-11-02

**Authors:** Rameshwar Dubey

**Affiliations:** 1 Montpellier Business School Montpellier Research in Management, 2300 Avenue des Moulins, 34185 Montpellier, France; 2 Liverpool Business School Liverpool John Moore’s University, Liverpool, Merseyside L3 5UG UK

**Keywords:** Humanitarian supply chain, Humanitarian operations management, Disaster relief operations, Humanitarian supply chain design

## Abstract

The design and management of the humanitarian supply chain are the most critical aspects of the humanitarian aid supply chain. Despite enormous interest among the academic community and the practitioners, the design of a humanitarian supply chain is still not well understood. Most of the publications have attempted to address the mechanisms of the humanitarian relief operations. However, the elements of the humanitarian supply chain designs are not well understood in an integrated manner. In this special issue, we have accepted the articles based on six factors that shape the design and management of the humanitarian supply chain and the influencing factors (see Fig. 4). We have noted the research gaps and offered rich directions for future research.

## Introduction

In the last decade, the humanitarian supply chain management field has gained significant attention from academics and policymakers (Kovacs & Spens, 2011a; Gupta et al., 2016; Altay et al., 2021). Humanitarian supply chain management has gained a significant footing after the 2004 Indian Tsunami. The disaster relief efforts following the 2004 Tsunami have received severe criticisms from experts due to poor supply chain management (Kovacs & Spens, 2011b; Oloruntoba et al., 2019). Since then, natural disasters are on the rise (Guha-Sapir & Scales, 2020). As more humanitarian crises are caused by disasters, supply chain research in humanitarian settings must continue to advance in such complex settings (Van Wassenhove, 2006; Starr & Van Wassenhove, 2014; Altay & Labonte, 2014). Following the definition of Burkart et al., (2016, p. 32), “*the process of planning, implementing and controlling the efficient, cost-effective flow and storage of goods and materials, as well as related information, from the point of origin to the point of consumption to alleviate the suffering of vulnerable people”*, we argue that the design and management of humanitarian supply chain design is one of the most critical aspects of the humanitarian supply chain management. Humanitarian organizations need to respond to the crises on an urgent basis, providing aid to the victims including shelter, food, and other necessary items to alleviate the sufferings of the victims (Charles et al., 2016). In the past, humanitarian organizations have acted in a way to gain maximum benefits in designing the humanitarian supply chain networks. The humanitarian organizations have either positioned their inventories in the location where they are engaged in the relief operations or closer to the airport or the location where they gain a maximum tax advantage (Pettit & Beresford, 2009; Roh et al., 2015). These approaches might have limited the scope of exploring other possible locational advantages (Charles et al., 2016). Thus, the design and management of an optimal supply chain network for the humanitarian organizations that operate in a highly complex setup is a major challenge for the humanitarian practitioners and the policymakers.

### Why focus on design and humanitarian supply chain?

**Fig. 1:**
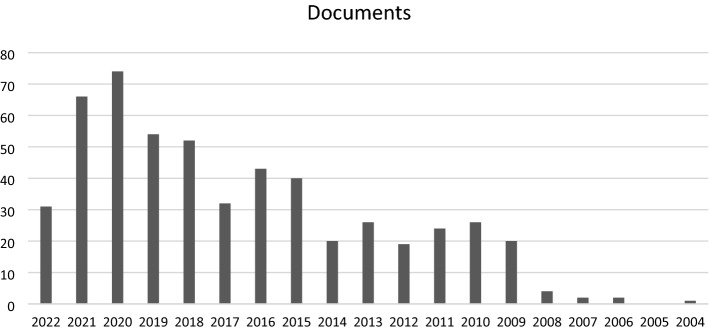
Publications by year (Humanitarian supply chain management)

**Fig. 2:**
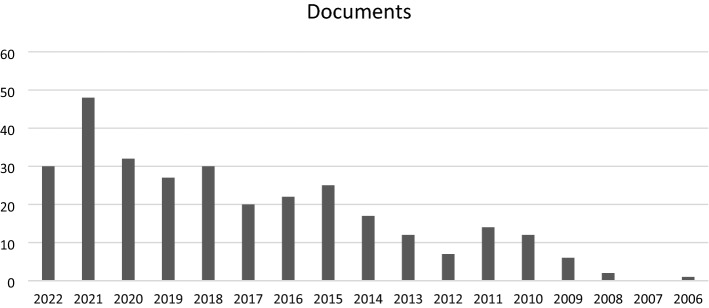
Publications by year (Humanitarian supply chain design)

In recent years the number of publications on humanitarian logistics/humanitarian supply chain management has increased significantly (see Fig. 1). Yet, few articles focusing on the humanitarian supply chain design suggest a significant research gap (see Fig. 2). Kovacs & Moshtari (2019) suggest that humanitarian studies should be more realistic and focus on real-world problems with a real data set. Charles et al., (2016) argue that the practitioners of humanitarian organizations find it difficult to grasp the underlying assumptions of the complex optimization problems. Moreover, in the case of robust and stochastic optimization, the practitioners drawn from the humanitarian organizations find it difficult to comprehend as it is often hard to assign the probabilities. Given these challenges, we followed the recommendation of Boyer & Swink (2008) that the multi-methods approach is the best way to tackle the complex challenges involved in the design and management of the humanitarian supply chain.

### The need for the special issue

**Fig. 3:**
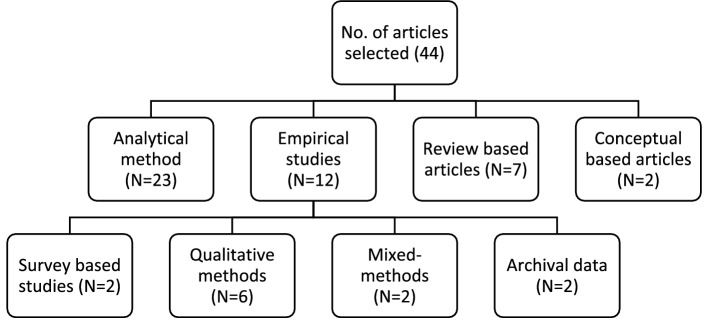
Classification scheme of the accepted articles (N = 44)

The special issue aimed to publish articles that will help advance the theoretical debates on how the humanitarian supply chain design can help tackle complex issues that often trouble the humanitarian relief workers during the disaster relief operations. The intent was clear to publish research articles that investigate the humanitarian supply chain design issues, identify various factors that influence the design and management of the humanitarian supply chain, and how these factors influence the humanitarian supply chain performance. There was no constraint on the type of submissions. These submissions could be analytical, conceptual, empirical studies relying on survey-based data, qualitative studies (i.e., multiple-case-based studies, action research, graph-theoretic approach, grounded theory, or ethnographic approach), or to an extent unique conceptual works that help push the theoretical boundary. Although, we encouraged the authors to address unique challenges faced by the humanitarian organizations in the wake of the exponential rise in disasters across the globe. The result was significant submissions of which we finally accepted 44 articles after multiple rounds of major revision. We have classified our accepted articles based on methods (see Fig. 3). Next, we provide the synthesis of the 44 contributions to theory and practice.

### Summary of contributions

**Fig. 4:**
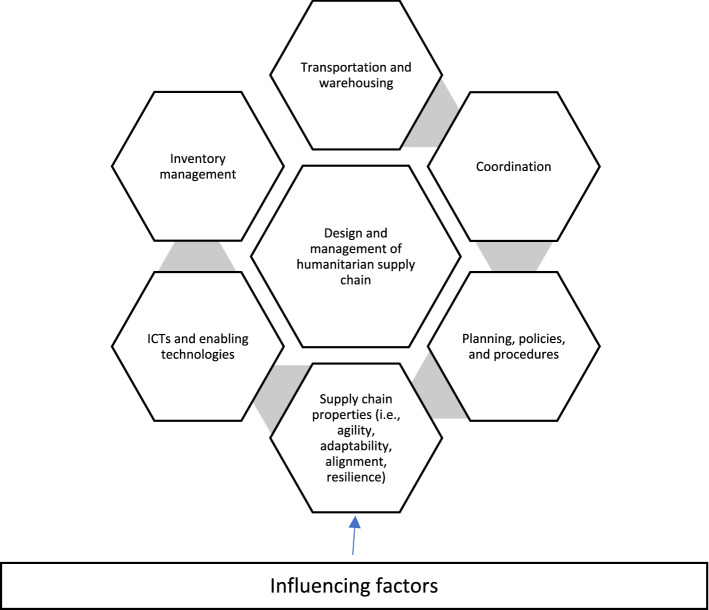
Factors of design and management of humanitarian supply chain

Before summarising the accepted contributions, however, it is important to understand them in the context of the design and management of the humanitarian supply chain. To begin with, we need to understand first what are the main factors that shape the humanitarian supply chain design (see Fig. 4).

**Table 1:** Summary of Literature

Factors	Contribution (see Appendix 1)	Findings of the study	Future research direction
Inventory management	A1, A5, A41	In A1 authors found that the options contract is one of the best ways of procuring the relief material. Whereas in A38 the authors propose an optimal solution to minimize the supply cost of the relief materials procured from various sources. A41 presents how the innovativeness of the suppliers helps tackle the complexity.	In the future, there is a significant scope to examine and evaluate other contracts to understand the implications of the procurement of relief materials.
Transportation and warehousing	A2, A3, A4, A5, A6, A7, A8, A9, A35, A36	The authors have attempted to provide optimal solutions using a wide range of options to improve flexibility, reduce cost and improve service during disaster relief operations.	Despite good efforts, there is a need for solutions that consider realistic conditions. There is a need for developing far more dynamic and robust techniques that help tackle realistic situations.
Coordination/collaboration	A10, A11, A1	The authors have discussed the role of coordination in reducing carbon emissions in a sustainable humanitarian supply chain (A10). Further, in A11 the authors have discussed the application of the technologies in improving coordination among the disaster relief actors in the I4.0 era. Further in article A1, the authors have examined the ways of improving coordination to improve the procurement strategy for relief items.	Despite some good efforts, the coordination among the disaster relief actors remains one of the most pressing concerns. In the future the scholars may pay detailed attention to governing mechanism. There is a need for a multi-method approach to building a comprehensive understanding of the coordination mechanism.
Planning, policies, and procedures	A12, A13, A14, A15, A37	The authors in article A12 argue beside the role of humanitarian assistance, the fund provided to the victims may help alleviate the suffering post-disaster phase. Further, the authors in article A13, proposed a unique method to evaluate the labor efficiency in the humanitarian sector. The authors in articles A14, A15, and A37 offer some implications for the policy to smoothen the disaster relief operations.	In the future how the humanitarian studies can shed more light based on empirical works on the policy front is called for. There is a need for a far more integrated approach to view the micro and macro elements that shape the humanitarian fabrics.
Supply chain properties	A16, A17, A18, A19, A20, A30	These articles contribute toward understanding agility, resilience, and how the ripple effect in the humanitarian supply chain can be understood.	In the future, there is a need for in-depth studies that may shed more insights on how to build agile and resilient humanitarian supply chains.
ICTs and enabling technologies	A21, A22, A23, A24, A25, A26, A27, A31	The role of AI-driven technologies, big data and predictive analytics, social media analytics, and other enabling technologies have received significant attention from humanitarian scholars. AI can facilitate the analysis and interpretation of vast and complex humanitarian datasets to improve decision-making (A21 and A23). Mobile applications, chatbots, and social media can create immediate feedback loops with people affected by humanitarian crises (A24, A25, and A27). Digital-enabled crowdfunding can offer a quick and flexible option (A23). In totality, these articles contribute to the body of knowledge of technology-enabled humanitarian relief action.	We understand well that despite multiple advantages, these technologies have their serious limitations. The technologies bring significant level challenges and risks. Inadequate data protection may pose a different level of risk, increase insecurities, and prevent coordinated humanitarian actions. Moreover, the lack of adequate IT infrastructure, poor access to technology, and digital literacy often leads to fragility and intensifies the exploitation of the weakest section of society. Incomplete datasets about affected people can lead to digital discrimination. Hence, there is a need for future studies that attempts to highlight the dark side of the technologies in the humanitarian relief action.
Influencing factors	A38, A39, A40, A41, A42, A43, A44	The authors offer multiple perspectives that may influence the design and management of the humanitarian supply chain that include forecasting capability, the displaced human beings, complexity, sustainability, pandemics, and culture.	In the future, humanitarian scholars can further examine the role of culture in the design of the humanitarian supply chain.

Further, we have also received publications that provide a retrospective outlook of the humanitarian supply chain management field. For instance, articles A28, A29, A30, A31, A32, A33, and A34 offer many insights to the humanitarian scholars and theories to test in future studies. For instance, article A28 has attempted to address the human-related issues in the humanitarian supply chain. Similarly, article A29 and A33 provides a retrospective review of the humanitarian supply chain literature published in reputable outlets and explains how the scholars and their scholarly output have shaped the evolution of humanitarian supply chain management as a discipline. The author points out some research gaps that may be worth investigating. The articles A30, A31, and A32 provide a detailed thematic review such as disruptions and resilience (A30), the role of digital technologies in the humanitarian supply chain (A31), and the quality management issues in the humanitarian supply chain (A32).

## Future research directions and opportunities

**Fig. 5:**
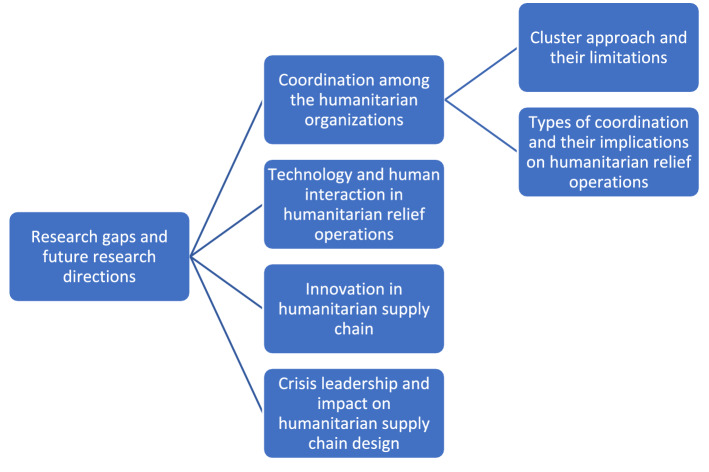
Research opportunities

One of the main aims of organizing this special issue was to identify potential research gaps and further motivate scholars to advance the theoretical debates surrounding humanitarian supply chain design. In Table 1, an attempt has been made to identify some research gaps, however, we believe that the gaps should not be limited to these research gaps noted in Table 1. It must help address the overall challenges that humanitarian organizations face while dealing with such unpredictable events with limited resources and are subject to a high level of scrutiny from the media and political organizations. Therefore, we provide a list of areas that can be tackled in future studies. For instance, the coordination among the humanitarian organizations has received significant attention from the humanitarian communities (see Balcik et al., 2010; Dubey et al., 2019; Ruesch et al., 2022). Yet, coordination in the humanitarian supply chain context is still not well understood. Future research should explore the fit between different types of coordination and humanitarian supply chain strategies. Secondly, following the Haiti earthquake, the use of technology in the humanitarian aid supply chain has received significant attention (Besiou & Van Wassenhove, 2020). However, still, the use of technology in the humanitarian aid supply chain faces enormous challenges (Dubey, 2022). The future study must help address the technology and human interaction issues. Thirdly, innovation in the humanitarian supply chain in recent times has played a significant role in tackling the most complex humanitarian crises (Kovács & Falagara Sigala, 2021). Yet, the innovation in the humanitarian supply chain is not well understood. We believe future studies must help address this research gap. Finally, the role of leadership has been recognized as an important driver in shaping the humanitarian aid supply chain (Salem et al., 2019; Dubey et al., 2021). However, the leadership styles differ in different situations. The humanitarian supply chain literature has largely remained silent on this front with some exceptions (see, Salem et al., 2018, 2019; Dubey, 2022). There is a clear research gap that needs to be addressed to understand how different leadership styles can help tackle complex humanitarian relief operations (Fig. 5).

## Concluding remarks

There is an enormous interest in the design and management of humanitarian supply chains among the operations and supply chain management community. Yet, the design of the humanitarian supply chain is not well understood. Hence, to bridge the potential gaps that exist in theory and practice, we accepted articles on various aspects of the humanitarian supply chain design and management (see Table 1). Further, we have noted some potential research gaps that may help future scholars to shape their future research.

## Appendix 1

- **A1**: John, L., Gurumurthy, A., Mateen, A., & Narayanamurthy, G. (2020). Improving the coordination in the humanitarian supply chain: Exploring the role of the options contract. *Annals of Operations Research*. DOI: 10.1007/s10479-020-03778-3.
- **A2**: Atsiz, E., Balcik, B., Gunnec, D., & Sevindik, B. U. (2021). A coordinated repair routing problem for post-disaster recovery of interdependent infrastructure networks. *Annals of Operations Research*. DOI: 10.1007/s10479-020-03909.
- **A3**: Farrokhizadeh, E., Seyfi-Shishavan, S. A., & Satoglu, S. I. (2021). Blood supply planning during natural disasters under uncertainty: a novel bi-objective model and an application for red crescent. *Annals of Operations Research*. DOI: 10.1007/s10479-021-03978-5.
- **A4**: Gao, X. (2019). A bi-level stochastic optimization model for multi-commodity rebalancing under uncertainty in disaster response. *Annals of Operations Research*. DOI: 10.1007/s10479-019-03506-6.
- **A5**: Jana, R. K., Sharma, D. K., & Mehta, P. (2021). A probabilistic fuzzy goal programming model for managing the supply of emergency relief materials. *Annals of Operations Research*. DOI: 10.1007/s10479-021-04267-x.
- **A6**: Khorsi, M., Chaharsooghi, S. K., Husseinzadeh Kashan, A., & Bozorgi-Amiri, A. (2022). Solving the humanitarian multi-trip cumulative capacitated routing problem via a grouping metaheuristic algorithm. *Annals of Operations Research*. DOI: 10.1007/s10479-022-04757-6.
- **A7**: Seraji, H., Tavakkoli-Moghaddam, R., Asian, S., & Kaur, H. (2021). An integrative location-allocation model for humanitarian logistics with distributive injustice and dissatisfaction under uncertainty. *Annals of Operations Research*. DOI: 10.1007/s10479-021-04003-5.
- **A8**: Stallkamp, C., Diehlmann, F., Lüttenberg, M., Wiens, M., Volk, R., & Schultmann, F. (2020). On the combination of water emergency wells and mobile treatment systems: a case study of the city of Berlin. *Annals of Operations Research*,. DOI: 10.1007/s10479-020-03800-8.
- **A9**: Lu, Y., Yang, C., & Yang, J. (2022). A multi-objective humanitarian pickup and delivery vehicle routing problem with drones. *Annals of Operations Research*. DOI: 10.1007/s10479-022-04816-y.
- **A10**: Fuli, G., Foropon, C., & Xin, M. (2020). Reducing carbon emissions in the humanitarian supply chain: The role of decision making and coordination. *Annals of Operations Research*. DOI: 10.10T07/s10479-020-03671-z.
- **A11**: Kumar, P., & Singh, R. K. (2021). Application of Industry 4.0 technologies for effective coordination in humanitarian supply chains: a strategic approach. *Annals of Operations Research*. DOI: 10.1007/s10479-020-03898-w.
- **A12**: Chen, J., Wang, P., Zhou, J., Song, M., & Zhang, X. (2020). Influencing factors and efficiency of funds in humanitarian supply chains: the case of Chinese rural minimum living security funds. *Annals of Operations Research*. DOI: 10.1007/s10479-020-03660-2.
- **A13**: Cheng, S., Fan, W., & Wang, J. (2020). Investigating the humanitarian labor efficiency of China: a factor-specific model. *Annals of Operations Research*. DOI: 10.1007/s10479-020-03736-z.
- **A14**: Haeri, A., Hosseini-Motlagh, S. M., Samani, M. R. G., & Rezaei, M. (2021). An integrated socially responsible-efficient approach toward health service network design. *Annals of Operations Research* DOI: 10.1007/s10479-021-04066-4.
- **A15**: Hakimifar, M., Balcik, B., Fikar, C., Hemmelmayr, V., & Wakolbinger, T. (2021). Evaluation of field visit planning heuristics during rapid needs assessment in an uncertain post-disaster environment. *Annals of Operations Research*. DOI: 10.1007/s10479-021-04274-y.
- **A16**: Dubey, R., Bryde, D. J., Foropon, C., Graham, G., Giannakis, M., & Mishra, D. B. (2020). Agility in humanitarian supply chain: An organizational information processing perspective and relational view. *Annals of Operations Research*. DOI: 10.1007/s10479-020-03824-0.
- **A17**: Hosseini, S., & Ivanov, D. (2019). A new resilience measure for supply networks with the ripple effect considerations: A Bayesian network approach. *Annals of Operations Research*, 1–27. DOI: 10.1007/s10479-019-03350-8.
- **A18**: Pavlov, A., Ivanov, D., Werner, F., Dolgui, A., & Sokolov, B. (2019). Integrated detection of disruption scenarios, the ripple effect dispersal and recovery paths in supply chains. *Annals of Operations Research*, 1–23. DOI: 10.1007/s10479-019-03454-1.
- **A19**: Stewart, M., & Ivanov, D. (2019). Design redundancy in agile and resilient humanitarian supply chains. *Annals of Operations Research*, 1–27. DOI: 10.1007/s10479-019-03507-5.
- **A20**: Mwangi, G. M., Despoudi, S., Espindola, O. R., Spanaki, K., & Papadopoulos, T. (2021). A planetary boundaries perspective on the sustainability: resilience relationship in the Kenyan tea supply chain. *Annals of Operations Research*, 1–35. DOI: 10.1007/s10479-021-04096-y.
- **A21**: Al Qundus, J., Dabbour, K., Gupta, S., Meissonier, R., & Paschke, A. (2020). Wireless sensor network for AI-based flood disaster detection. *Annals of Operations Research*, 1–23. DOI: 10.1007/s10479-020-03754-x.
- **A22**: Bag, S., Gupta, S., & Wood, L. (2020). Big data analytics in sustainable humanitarian supply chain: Barriers and their interactions. *Annals of Operations Research*, 1–40. DOI: 10.1007/s10479-020-03790-7.
- **A23**: Behl, A., Dutta, P., Luo, Z., & Sheorey, P. (2021). Enabling artificial intelligence on a donation-based crowdfunding platform: a theoretical approach. *Annals of Operations Research*, 1–29. DOI: 10.1007/s10479-020-03906-z.
- **A24**: Kumar, A., Singh, J. P., Dwivedi, Y. K., & Rana, N. P. (2020). A deep multi-modal neural network for informative Twitter content classification during emergencies. *Annals of Operations Research*, 1–32. DOI: 10.1007/s10479-020-03514-x.
- **A25**: Kumar, S., Xu, C., Ghildayal, N., Chandra, C., & Yang, M. (2021). Social media effectiveness as a humanitarian response to mitigate the influenza epidemic and COVID-19 pandemic. *Annals of Operations Research*, 1–29. DOI: 10.1007/s10479-021-03955-y.
- **A26**: Mishra, S., & Singh, S. P. (2020). A stochastic disaster-resilient and sustainable reverse logistics model in big data environment. *Annals of Operations Research*, 1–32. DOI: 10.1007/s10479-020-03573-0.
- **A27**: Nagendra, N. P., Narayanamurthy, G., & Moser, R. (2020). Management of humanitarian relief operations using satellite big data analytics: The case of Kerala floods. *Annals of operations research*, 1–26. DOI: 10.1007/s10479-020-03593-w.
- **A28**: de Camargo Fiorini, P., Chiappetta Jabbour, C. J., Lopes de Sousa Jabbour, A. B., & Ramsden, G. (2021). The human side of humanitarian supply chains: a research agenda and systematization framework. *Annals of Operations Research*, 1–26. DOI: 10.1007/s10479-021-03970-z.
- **A29**: Fosso Wamba, S. (2020). Humanitarian supply chain: A bibliometric analysis and future research directions. *Annals of Operations Research*, 1–27. DOI: 10.1007/s10479-020-03594-9.
- **A30**: Katsaliaki, K., Galetsi, P., & Kumar, S. (2021). Supply chain disruptions and resilience: A major review and future research agenda. *Annals of Operations Research*, 1–38. DOI: 10.1007/s10479-020-03912-1.
- **A31**: Marić, J., Galera-Zarco, C., & Opazo-Basáez, M. (2021). The emergent role of digital technologies in the context of humanitarian supply chains: a systematic literature review. *Annals of Operations Research*, 1–42. DOI: 10.1007/s10479-021-04079-z.
- **A32**: Modgil, S., Singh, R. K., & Foropon, C. (2020). Quality management in humanitarian operations and disaster relief management: A review and future research directions. *Annals of Operations Research*, 1–54. DOI: 10.1007/s10479-020-03695-5.
- **A33**: Nunes, R. M. S., & Pereira, S. C. F. (2021). Intellectual structure and trends in the humanitarian operations field. *Annals of Operations Research*, 1–59. DOI: 10.1007/s10479-021-04022-2.
- **A34**: Queiroz, Maciel M., Dmitry Ivanov, Alexandre Dolgui, and Samuel Fosso Wamba. Impacts of epidemic outbreaks on supply chains: mapping a research agenda amid the COVID-19 pandemic through a structured literature review. *Annals of Operations Research* (2020): 1–38. DOI: 10.1007/s10479-020-03685-7.
- **A35**: Baharmand, H., Vega, D., Lauras, M., & Comes, T. (2022). A methodology for developing evidence-based optimization models in humanitarian logistics. *Annals of Operations Research*, 1–33. DOI: 10.1007/s10479-022-04762-9.
- **A36**: Chang, Y., Song, Y., & Eksioglu, B. (2021). A stochastic look-ahead approach for hurricane relief logistics operations planning under uncertainty. *Annals of Operations Research*, 1–33. DOI: 10.1007/s10479-021-04025-z.
- **A37**: Jamali, A., Ranjbar, A., Heydari, J., & Nayeri, S. (2021). A multi-objective stochastic programming model to configure a sustainable humanitarian logistics considering deprivation cost and patient severity. *Annals of operations research*, 1–36. DOI: 10.1007/s10479-021-04014-2.
- **A38**: Paul, S., & Davis, L. B. (2021). An ensemble forecasting model for predicting contribution of food donors based on supply behavior. *Annals of operations research*, 1–29. DOI: 10.1007/s10479-021-04146-5.
- **A39**: Prasad, S., Woldt, J., Borra, H., & Altay, N. (2020). Migrant supply chain networks: an empirically based typology. *Annals of Operations Research*, 1–28. DOI: 10.1007/s10479-020-03523-w.
- **A40**: Prasanna, S. R. (2021). The role of supplier innovativeness in the humanitarian context. *Annals of Operations Research*, 1–19. DOI: 10.1007/s10479-021-04065-5.
- **A41**: Schiffling, S., Hannibal, C., Tickle, M., & Fan, Y. (2020). The implications of complexity for humanitarian logistics: A complex adaptive systems perspective. *Annals of Operations Research*, 1–32. DOI: 10.1007/s10479-020-03658-w.
- **A 42**: Ivanov, D. (2020). Viable supply chain model: integrating agility, resilience and sustainability perspectives—lessons from and thinking beyond the COVID-19 pandemic. *Annals of operations research*, 1–21.DOI: 10.1007/s10479-020-03640-6.
- **A43**: Gupta, M., Shoja, A., & Mikalef, P. (2021). Toward the understanding of national culture in the success of non-pharmaceutical technological interventions in mitigating COVID-19 pandemic. *Annals of Operations Research*, 1–18. DOI: 10.1007/s10479-021-03962-z.
- **A44**: Shareef, M. A., Dwivedi, Y. K., Kumar, V., Hughes, D. L., & Raman, R. (2020). Sustainable supply chain for disaster management: Structural dynamics and disruptive risks. *Annals of Operations Research*, 1–25. DOI: 10.1007/s10479-020-03708-3.

## References

[CR1] Altay N, Labonte M (2014). Challenges in humanitarian information management and exchange: Evidence from Haiti. Disasters.

[CR2] Altay N, Kovács G, Spens K (2021). The evolution of humanitarian logistics as a discipline through a crystal ball. Journal of Humanitarian Logistics and Supply Chain Management.

[CR3] Balcik B, Beamon BM, Krejci CC, Muramatsu KM, Ramirez M (2010). Coordination in humanitarian relief chains: Practices, challenges and opportunities. International Journal of Production Economics.

[CR4] Besiou M, Van Wassenhove LN (2020). Humanitarian operations: A world of opportunity for relevant and impactful research. Manufacturing & Service Operations Management.

[CR5] Boyer KK, Swink ML (2008). Empirical elephants—Why multiple methods are essential to quality research in operations and supply chain management. Journal of Operations Management.

[CR6] Burkart C, Besiou M, Wakolbinger T (2016). The funding—Humanitarian supply chain interface. Surveys in Operations Research and Management Science.

[CR7] Charles A, Lauras M, Van Wassenhove LN, Dupont L (2016). Designing an efficient humanitarian supply network. Journal of Operations Management.

[CR8] Dubey R, Altay N, Blome C (2019). Swift trust and commitment: The missing links for humanitarian supply chain coordination?. Annals of Operations Research.

[CR9] Dubey R, Bryde DJ, Foropon C, Tiwari M, Dwivedi Y, Schiffling S (2021). An investigation of information alignment and collaboration as complements to supply chain agility in humanitarian supply chain. International Journal of Production Research.

[CR10] Dubey R (2022). Unleashing the potential of digital technologies in emergency supply chain: The moderating effect of crisis leadership. Industrial Management & Data Systems.

[CR11] Guha-Sapir D, Scales SE (2020). Challenges in public health and epidemiology research in humanitarian settings: Experiences from the field. Bmc Public Health.

[CR12] Gupta S, Starr MK, Farahani RZ, Matinrad N (2016). Disaster management from a POM perspective: Mapping a new domain. Production and Operations Management.

[CR13] Kovács G, Spens KM (2011). Trends and developments in humanitarian logistics–a gap analysis. International Journal of Physical Distribution & Logistics Management.

[CR14] Kovács G, Spens K (2011). Humanitarian logistics and supply chain management: The start of a new journal. Journal of Humanitarian Logistics and Supply Chain Management.

[CR15] Kovács G, Moshtari M (2019). A roadmap for higher research quality in humanitarian operations: A methodological perspective. European Journal of Operational Research.

[CR16] Kovács G, Falagara Sigala I (2021). Lessons learned from humanitarian logistics to manage supply chain disruptions. Journal of Supply Chain Management.

[CR17] Oloruntoba R, Hossain GF, Wagner B (2019). Theory in humanitarian operations research. Annals of Operations Research.

[CR18] Pettit SJ, Beresford AKC (2009). Critical success factors in the context of humanitarian aid supply chains. International Journal of Physical Distribution & Logistics Management.

[CR19] Ruesch L, Tarakci M, Besiou M, Van Quaquebeke N (2022). Orchestrating coordination among humanitarian organizations. Production and Operations Management.

[CR20] Roh S, Pettit S, Harris I, Beresford A (2015). The pre-positioning of warehouses at regional and local levels for a humanitarian relief organization. International Journal of Production Economics.

[CR21] Salem M, Van Quaquebeke N, Besiou M (2018). How field office leaders drive learning and creativity in humanitarian aid: Exploring the role of boundary-spanning leadership for expatriate and local aid worker collaboration. Journal of Organizational Behavior.

[CR22] Salem M, Van Quaquebeke N, Besiou M, Meyer L (2019). Intergroup leadership: How leaders can enhance performance of humanitarian operations. Production and Operations Management.

[CR23] Schiffling S, Hannibal C, Fan Y, Tickle M (2020). Coopetition in temporary contexts: Examining swift trust and swift distrust in humanitarian operations. International Journal of Operations and Production Management.

[CR24] Starr MK, Van Wassenhove LN (2014). Introduction to the special issue on humanitarian operations and crisis management. Production and Operations Management.

[CR25] Van Wassenhove LN (2006). Humanitarian aid logistics: supply chain management in high gear. Journal of the Operational Research Society.

